# Establishment of a nonshockable rhythm cardiac arrest model caused by asphyxia

**DOI:** 10.1186/s12872-022-02996-w

**Published:** 2022-12-29

**Authors:** Chenyu Zhang, Haohong Zhan, Dawang Zhou, Tian Li, Qiang Zhang, Cong Liu, Hongyan Wei, Chunlin Hu

**Affiliations:** 1 Department of Emergency Medicine, The First Affiliated Hospital of Sun Yat-sen University, The 58 Zhongshan Er Rd, Guangzhou, 510080 People’s Republic of China; 2 Department of Critical Care Medicine, The First Affiliated Hospital of Sun Yat-sen University, The 58 Zhongshan Er Rd, Guangzhou, 510080 People’s Republic of China; 3 NHC Key Laboratory of Assisted Circulation, Sun Yat-sen University, Guangzhou, 510080 People’s Republic of China; 4 Department of Emergency Medicine, The Seventh affiliated hospital of Sun Yat-sen University, 628, Zhenyuan Rd, Guangming (New) Dist, Shenzhen, 518107 People’s Republic of China; 5 School of Basic Medicine, Fourth Military Medical University, 169 Changle West Rd, Xi’an, 710032 People’s Republic of China

**Keywords:** Cardiac arrest, Nonshockable rhythm, Cardiac function, Neurologic impairment

## Abstract

**Objective:**

Cardiac arrest (CA) is caused by a nonshockable rhythm with a low success rate of return of spontaneous circulation (ROSC) and a poor prognosis. This study intended to establish a nonshockable rhythm CA model caused by asphyxia.

**Materials and methods:**

Healthy adult male Wistar rats were injected with vecuronium bromide to induce CA. After the CA duration reached the target time point, cardiopulmonary resuscitation was performed. The survival status and neurological and cardiac function were evaluated after ROSC. Brain histopathology, including hematoxylin staining, Nissl staining and Terminal dUTP nick-end labeling (TUNEL) staining, was performed to evaluate the surviving cells and apoptotic cells. Apoptosis-related proteins after ROSC for 72 h were analyzed by western blot.

**Results:**

CA was successfully induced in all animals. The time for the three groups of animals to PEA was 320 ± 22 s in the CA-8 group, 322 ± 28 s in the CA-12 group and 320 ± 18 s in the CA-15 group. The time to asystole was 436 ± 54 s in the CA-8 group, 438 ± 62 s in the CA-12 group and 433 ± 56 s in the CA-15 group. The NDS of rats in the CA group was significantly decreased after ROSC for 24 h. The NDS in the CA-15 group was 5–16 points, while it was 58–67 points and 15–43 points in the CA-8 and CA-12 groups, respectively. The cardiac function of animals in the CA group was impaired after ROSC, and the ejection fraction, fractional shortening, stroke volume and cardiac output, were all significantly decreased. Brain histopathology showed that the number of surviving neurons was decreased, and the number of apoptotic cells was increased in CA group, the longer the CA duration, the more apoptotic cells increased. The expression of the proapoptotic protein Bax and the apoptotic executive protein caspase3 in the hippocampus of CA rats was significantly increased, while the expression of the antiapoptotic protein Bcl-2 was significantly reduced.

**Conclusions:**

The use of vecuronium can successfully induce CA caused by nonshockable rhythm in rats, which will help to further study the pathophysiological changes after CA by nonshockable rhythm.

**Supplementary Information:**

The online version contains supplementary material available at 10.1186/s12872-022-02996-w.

## Introduction

Cardiac arrest (CA) refers to the cessation of mechanical activity of the heart and the disappearance of circulatory signs. It is one of the main causes of death in the world and one of the highest mortality diseases in China, causing hundreds of thousands of deaths each year [1]. Although the mortality rate of cardiac arrest is very high, it has received little attention compared with other high-risk cardiovascular diseases, such as stroke and myocardial infarction. Initial heart rhythm and return of spontaneous circulation (ROSC) time are the two most relevant factors for patient survival [2]. Different heart diseases induce different rhythms of CA, and their treatment and prognosis are also very different. According to the electrical and mechanical activities of the heart, the onset of CA can be divided into “shockable rhythm”, including ventricular fibrillation (VF), pulseless ventricular tachycardia (PVT), and “nonshockable heart rhythms”, including pulseless electrical activity (PEA) and asystole. However, there are also opinions that PEA and asystole are two different concepts, and the two types of patients have different characteristics before and during CA [3, 4]. Compared with PEA patients, the prognosis of asystole patients tends to be worse [5].

Animal models have been widely used in the study of CA. The most commonly used animals are pigs (52%), followed by rats (35%) and mice (6%), and researchers prefer to use male animals (52%) [6]. The most common methods of inducing CA are electrically induced VF (54%), asphyxia (25%) or potassium (8%). The time of no blood flow is usually 8 min, mainly concentrated in 6–15 min [7–9]. There is huge heterogeneity among various animal models of CA, which makes it difficult to compare different studies. The guidelines recommend the use of the Utstein animal report template for laboratory animals to facilitate comparisons between different studies [10].

The CA-induced ventricular fibrillation CPR animal model used by our team in a previous study [11–13] was combined with defibrillation, cardiopulmonary resuscitation (CPR) technology, and epinephrine to obtain ROSC. However, there were large differences in the number of defibrillations, ROSC time, and epinephrine dosage between animals.

The ultimate purpose of animal models is to reflect the actual clinical situation, thereby transforming research results into clinical applications. However, most animal models fail to reflect the actual clinical situation, so many research results cannot be translated into clinical practice [14]. Electrically induced ventricular fibrillation represents the shockable rhythm CA, while asphyxia- or potassium-induced CA represents the nonshockable rhythm CA. The treatment methods and success rates of the two are very different [2]. The treatment of CA caused by nonshockable rhythm is difficult, and the success rate is low, which should attract the attention of clinicians. This study intends to establish a rat CPR model of CA caused by nonshockable rhythm, which will help to further study the pathophysiological changes after CA caused by nonshockable rhythm and provide clues for clinicians to address these challenges.

## Materials and methods

### Animal preparation, CA model and CPR

Fifty healthy male Wistar rats weighing 349–415 g were purchased from the Experimental Animal Center of Southern Medical University. All rats were allowed to eat and drink freely before and after the experiment and were housed in a quiet environment with a 12-hour day-night cycle. All rats were adapted for 4 weeks before the experiment. All rats were fasted overnight for 12 h before the experiment and drank freely. After fasting overnight, the animals were anesthetized by intraperitoneal injection of 3% sodium pentobarbital (Sigma, St. Louis, MO, USA) 45 mg/kg. When the rat was completely unconscious, a 14G tracheal tube was inserted, and a ventilator was used for mechanical ventilation during CPR. The right femoral artery was punctured for arterial blood pressure monitoring, the right femoral vein was punctured for intravenous administration, and the tube was sealed with heparin (2.5 IU/mL). An ECG monitor (PHILIPS SureSigns VM4, MA, USA) continuously recorded ECG, blood pressure, heart rate, respiratory rate, and SPO2. A ventilator (Harvard Rodent Ventilator; Harvard Apparatus, Holliston, MA) provided ventilation, at tidal volume 0.8 ml/100 g, respiratory rate 55/min, SPO2 > 93%. Ventilation parameters were adjusted using arterial blood gas results to maintain the PCO2 in the range of 35 mm Hg to 45 mm Hg.

For initiation of CA, Vecuronium bromide (0.1 mg/kg, IV) was given to block the animal’s breathing. The animal’s blood pressure gradually decreased due to hypoxia. When the mean arterial pressure (MAP) was lower than 30 mmHg, it was defined as CA. According to the duration of CA, this experiment was divided into the CA-8 min group, CA-12 min group and CA-15 min group. After the CA duration reached the target time point, CPR was performed, mechanical ventilation was resumed, epinephrine (20 µg/kg/3 min) and sodium bicarbonate (1.0 ml/kg) were intravenously injected, and the compression device developed by our laboratory was used to perform compression at a speed of 200 times/min. The depth of compression was 1/3 of the anteroposterior diameter of the thorax. ROSC was defined as the occurrence of supraventricular rhythm and MAP above 65 mmHg for more than 15 min. After ROSC, mechanical ventilation was continued, and the vital signs of the rats were monitored. If the ROSC was not reached after continuous resuscitation for more than 10 min, it was defined as ROSC failure (Additional file 2).

### Experimental design and grouping

**Fig. 1:**
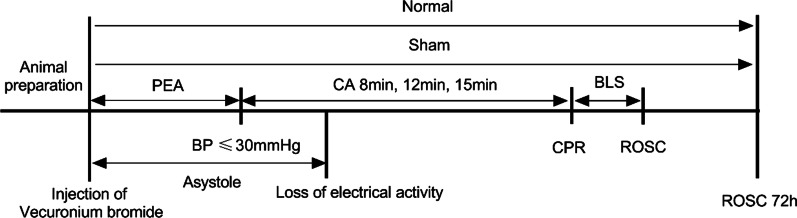
Flow chart of experiment. PEA, Pulseless electrical activity; ROSC, Return of spontaneous circulation; BLS, Base life support; CPR, Cardiopulmonary resuscitation; CA, Cardiac arrest; BP, Blood pressure. It was defined as CA When the BP was lower than 30 mmHg

ftablkeBased on the pre-experiments, we predict the number of each group by the success rate of animal models. Sixty-nine rats were randomly divided into 5 groups: (1) In the normal operation group (Normal control group), except for CA and CPR, the rest were given the same treatment until the end of the operation; (2) In the sham operation group (Sham group), the animals received the same treatment, but the injected muscles were relaxed and mechanical ventilation was performed at the same time as drug administration, (3) CA-8 min group, (4) CA-12 min group, (5) CA-15 min group. The relevant time definitions in this study were as follows: the time interval from when the muscle relaxant drug was injected to the MAP going below 30 mmHg was defined as the induction time; the time interval from the beginning of the muscle relaxant drug injection to MAP going below 30 mmHg and ECG performance displaying asystole was defined as PEA, and the time interval from the appearance of asystole to the beginning of CPR was defined as the asystole time. The time from the start of CPR to ROSC was defined as the base life support (BLS) time. The specific process is shown in Fig. 1.

### Survival status and neurological function assessment

The survival status of rats within 72 h after ROSC was recorded, and a survival curve was generated. The neurological deficit score (NDS) was used to evaluate the recovery of neurological function in rats [9]. The score included 7 aspects of wakefulness, brainstem function, motor function assessment, sensory function assessment, motor behavior, behavior and seizures. The NDS of a normal rat was 80 points, and a score of 0 indicated death. Neurological function was evaluated by independent investigators at three time points: 24 h, 48 and 72 h after ROSC. The total scores at different time points were used as independent evaluation indicators, and the 72-hour overall NDS was defined as the main end point of the neurological outcome.

### Echocardiographic evaluation

Under normal conditions and 4 h after ROSC, heart function was checked by echocardiography. The rats were anesthetized by inhalation of 3% isoflurane and maintained at a body temperature of 37 °C. A Vevo-2100 high-frequency ultrasound system (VisualSonics Inc., Toronto, Canada) was used to perform transechocardiography to evaluate left ventricular ejection fraction (EF), shortening fraction (FS), stroke volume (SV) and cardiac output (CO).

### Hematoxylin-eosin staining and Nissl Staining

The CA1 area of the hippocampus is a fragile area that is sensitive to cerebral ischemia and hypoxia and is a window commonly used to observe brain injury. Seventy-two hours after ROSC, the rats were sacrificed by deep anesthesia, and brain tissues were collected. Then, 150 ml of 0.01 mol/L PBS was perfused and washed from the apex of the heart which was fixed by perfusion with 4% paraformaldehyde. The brain was removed and divided into two halves along the sagittal plane. The two halves of the brain were immediately placed in a 4% paraformaldehyde solution at 4 °C, fixed, dehydrated, and embedded in paraffin before sectioning. The fixed heart and brain tissues were made into 5 micron-thick sections and then stained using a standard HE staining kit (Servicebio, Wuhan, China). The histology of the rat brain was observed under a microscope.

A Nissl staining kit (Servicebio, Wuhan, China) was used for staining, and the paraffin sections were deparaffinized and stained with toluidine blue solution (Beyotime Institute of Biotechnology, Nantong, China). Then, the cells were stained with 0.5% eosin for 3 s and rinsed under running water. Finally, Nissl bodies were identified under an optical microscope, and 6 fields of the hippocampal CA1 area were randomly selected for counting of cells positive for Nissl staining.

### TUNEL staining to identify apoptotic neuronal cells

According to the manufacturer’s instructions, an in-situ apoptosis detection kit (Nanjing Jiancheng Institute of Bioengineering, China) was used for TUNEL staining of brain sections collected 72 h after ROSC. First, the slices were placed in an oven at 60 °C for 60 min and then immersed in PBS for 5 min. Then, the sections were incubated in xylene at room temperature for 20 min and rinsed with PBS 3 times. The slides were incubated in permeation solution (0.3% Triton X-100, 0.1% sodium citrate) for 10 min. The TUNEL reaction mixture was added to each slice. Then, the cells were incubated for 60 min at 37 °C in a dark and humid environment. Finally, slices were prepared using anti-fluorescence attenuation mounting tablets containing DAPI. Three fields were randomly selected for each section, and TUNEL-positive cells were counted. Image-ProPlus 6.0 software (MediaCybernetics, Rockville, MD, USA) was used to calculate the percentage of apoptotic cells relative to the total number of cells.

### Western blot analysis of apoptosis-related proteins after ROSC for 72 h

In brief, the tissue was homogenized with a protein extraction reagent containing 1 mM PMSF. The supernatant was collected by centrifugation at 4 °C and 10,000 rpm for 30 min, and the protein concentration was measured by the BCA method. The protein was separated by SDS–PAGE and transferred to PVDF membranes, blocked in 5% skim milk for 2 h, and then incubated with primary antibodies against caspase 3 (1:2000 dilution; CST, USA), Bcl2 (1:2000 dilution; CST, USA), Bax (1:2000 dilution; CST, USA) and GAPDH (1:2000 dilution; CST, USA) overnight. After washing in TBST, the membrane was incubated with goat anti-rabbit antibody (diluted 1:5000; Abcam) in TBST for 1 h at room temperature. Finally, an electrochemiluminescence western blot system was used to detect specific bands. ImageJ software (National Institutes of Health, Bethesda, Maryland, USA) was used to analyze the density of immunoreactive membranes.

### Statistical analysis

All statistical analyses were performed using SPSS for Windows, version 23.0 (SPSS, Chicago, IL). All data were expressed as the mean ± standard deviation (SD), the comparison of two sample means was performed by t test, the comparison between data of more than three groups was performed by one-way analysis of variance (ANOVA), and multiple comparisons were performed by post hoc test. Nonnormally distributed data were analyzed by nonparametric tests, and the Mann–Whitney test was used to compare the NDS between groups. The ROSC rate was evaluated using the chi-square test. Kaplan–Meier survival analysis compared the differences in survival status between groups. *P* < 0.05 indicated that the difference was significant.

## Results

### The physiological parameters and CPR parameters of rats in each group

There was no significant difference in the basic physiological parameters of animals between the groups (Table 1). CA was successfully induced in all animals. The time for the three groups of animals to induce CA was 320 ± 22 s in the CA-8 group, 322 ± 28 s in the CA-12 group and 320 ± 18 s in the CA-15 group. The time to PEA after CA was successfully induced was 116 ± 32 s in the CA-8 group, 116 ± 34 s in the CA-12 group and 113 ± 38 s in the CA-15 group. BLS time and adrenaline dosage were significantly different between the groups (Table 1). There was no significant difference in the MAP of the animals in each group before CA, and the MAP between the animals in the groups after ROSC was significantly different (Table 1). It was necessary to use adrenaline to maintain MAP higher than 65 mmHg. There was no significant difference in ejection fraction (EF), left ventricular fraction (FS), stroke volume (SV), cardiac output (CO), or baseline values between the Normal group and the Sham group. The cardiac function of animals in the CA group was impaired after ROSC, the EF, FS, SV and CO were all significantly decreased, and there were significant differences between the three groups (Table 2).

**Table 1:** The physiological parameters and CPR parameters of rat in each group

Group	Normal	Sham	CA-8	CA-12	CA-15
n	6	6	6	12	39
BW(g)	379 ± 23	377 ± 21	382 ± 15	384 ± 21	377 ± 24
HR (Beats/min)	380 ± 31	364 ± 34	368 ± 30	377 ± 43	371 ± 39
SP (mmHg)	146 ± 15	133 ± 5	134 ± 17	143 ± 12	140 ± 12
DP (mmHg)	133 ± 16	121 ± 8	122 ± 17	130 ± 9	126 ± 13
MAP (mmHg)	140 ± 15	126 ± 6	129 ± 16	137 ± 10	134 ± 12
PEA (s)	0	0	320 ± 22	322 ± 28	320 ± 18
Asystole (s)	0	0	436 ± 54	438 ± 62	433 ± 56
BLS time (s)	0	0	99 ± 58	145 ± 54*	207 ± 107*#
Ventricular fibrillation (%)	0	0	0	0	0
Adrenaline dosage (µg)	0	0	0.4 ± 0.18	0.7 ± 0.45*	1.1 ± 0.49*#

*Compared with group CA-8, *P* < 0.05; #, compared with group CA-12, *P* < 0.05. *BW*, body weight, *HR*, heart rate, *SP*, systolic pressure, *DP*, diastolic pressure, *MAP*, mean arterial pressure, *PEA*, Pulseless electrical activity, *BLS*, base life support

**Table 2:** The cardiac function of animals at Basal and after ROSC 4 h in each group

Group	Normal	Sham	CA-8	CA-12	CA-15	*P* value
*Basal*						
LVEF (%)	94 ± 3	90 ± 2.5	92 ± 1.4	92 ± 2.9	94 ± 4.4	0.52
FS (%)	70 ± 5.8	63 ± 3.9	66 ± 2.3	66 ± 4.6	71 ± 9.1	0.43
SV (ul)	230 ± 30	236 ± 36	230 ± 29	242 ± 21	240 ± 33	0.96
CO (ml/min)	90 ± 25	102 ± 10	94 ± 9	98 ± 22	94 ± 18	0.93
*ROSC 4 h*						
LVEF (%)	94 ± 2.2	96 ± 2.4	79 ± 2.6	72 ± 5.2*	36 ± 10.9*#	< 0.01
FS (%)	70 ± 4.3	74 ± 5.6	49 ± 2.2	41 ± 4.5*	17 ± 5.6*#	< 0.01
SV (ul)	194 ± 44	169 ± 48	101 ± 17	76 ± 14*	12 ± 4.9*#	< 0.01
CO (ml/min)	79 ± 15	68 ± 18	40 ± 5	26 ± 4*	5 ± 1*#	< 0.01

*Compared with group CA-8, *P* < 0.05; #, compared with group CA-12, *P* < 0.05. *LVEF*, left ventricular ejection fraction, *FS*, shortening fraction, *SV*, stroke volume, *CO*, cardiac output

### Analysis of the ROSC rate and KM survival status of rats in each group

**Fig. 2:**
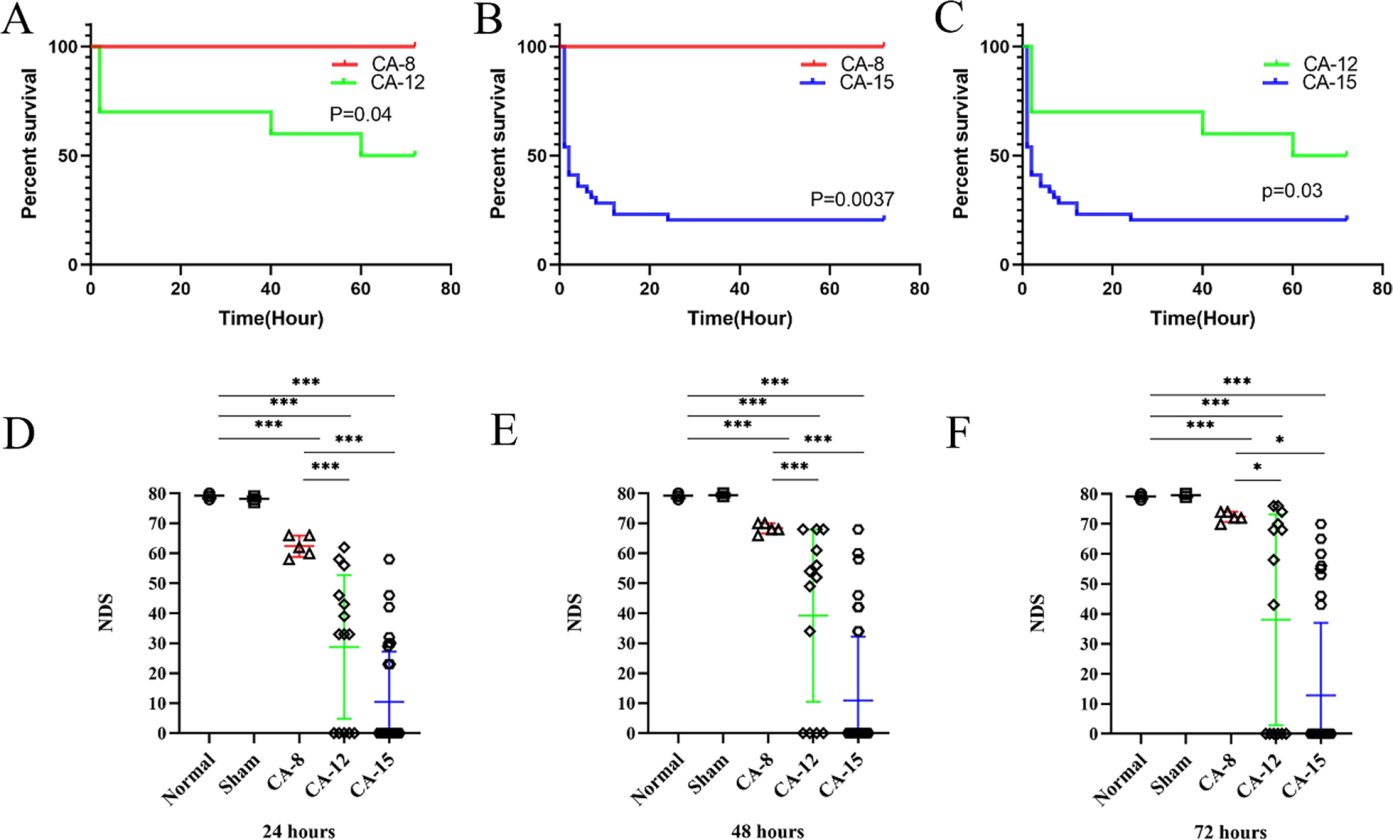
The survival status and neurological deficit score of rats in each group after ROSC. **A** The survival status of rats in the CA-8 group was better than that of rats in the CA-12 group. **B** The survival status of rats in the CA-8 group was better than that in the CA-15 group. **C** The survival status of rats in the CA-12 group was better than that in the CA-15 group. **D** The NDS of rats in the CA group was significantly decreased after ROSC for 24 h, and the NDS was 5–16 points in the CA-15 group, 58–67 points in the CA-8 group and 15–43 points in the CA-12 group. **E** The NDS of animals in each group after ROSC for 48 h. **F** The NDS of animals in each group after ROSC for 72 h. *P < 0.05; **P < 0.01; ***P < 0.001

To ensure animal welfare and reduce unnecessary waste, we only included 5 animals in the Normal group and the Sham group and found that their survival rate within 72 h after surgery was 100%. The ROSC rate of rats in the CA-8 group was 100% (6/6), and the survival rate at 72 h was 100% (6/6); the ROSC rate of rats in the CA-12 group was 100%, and the survival rate at 72 h was 58% (7/12). The ROSC rate of rats in the CA-15 group was 56% (22/39), and the 72 h survival rate was only 21% (8/39). There were significant differences in the ROSC rate among the groups. Kaplan–Meier survival curves showed that the survival rate of rats in the CA-15 group dropped sharply within 24 h after ROSC, where 14 animals died of refractory cardiogenic shock, only 3 in the CA-12 group died of refractory cardiogenic shock, and none died of this condition in the CA-8 group. The survival status within 72 h was significantly different between the groups (Fig. 2A–C).

The rats in the normal group and the sham group were not found to have neurological deficit symptoms. The NDS of rats in the CA group was significantly decreased after ROSC for 24 h. The NDS in the CA-15 group was 5–16 points, while it was 58–67 points and 15–43 points in the CA-8 and CA-12 groups, respectively. With the extension of ROSC time, the animal’s NDS gradually improved, but there were still significant differences between the groups (Fig. 2D–F).

### Brain histopathology

**Fig. 3:**
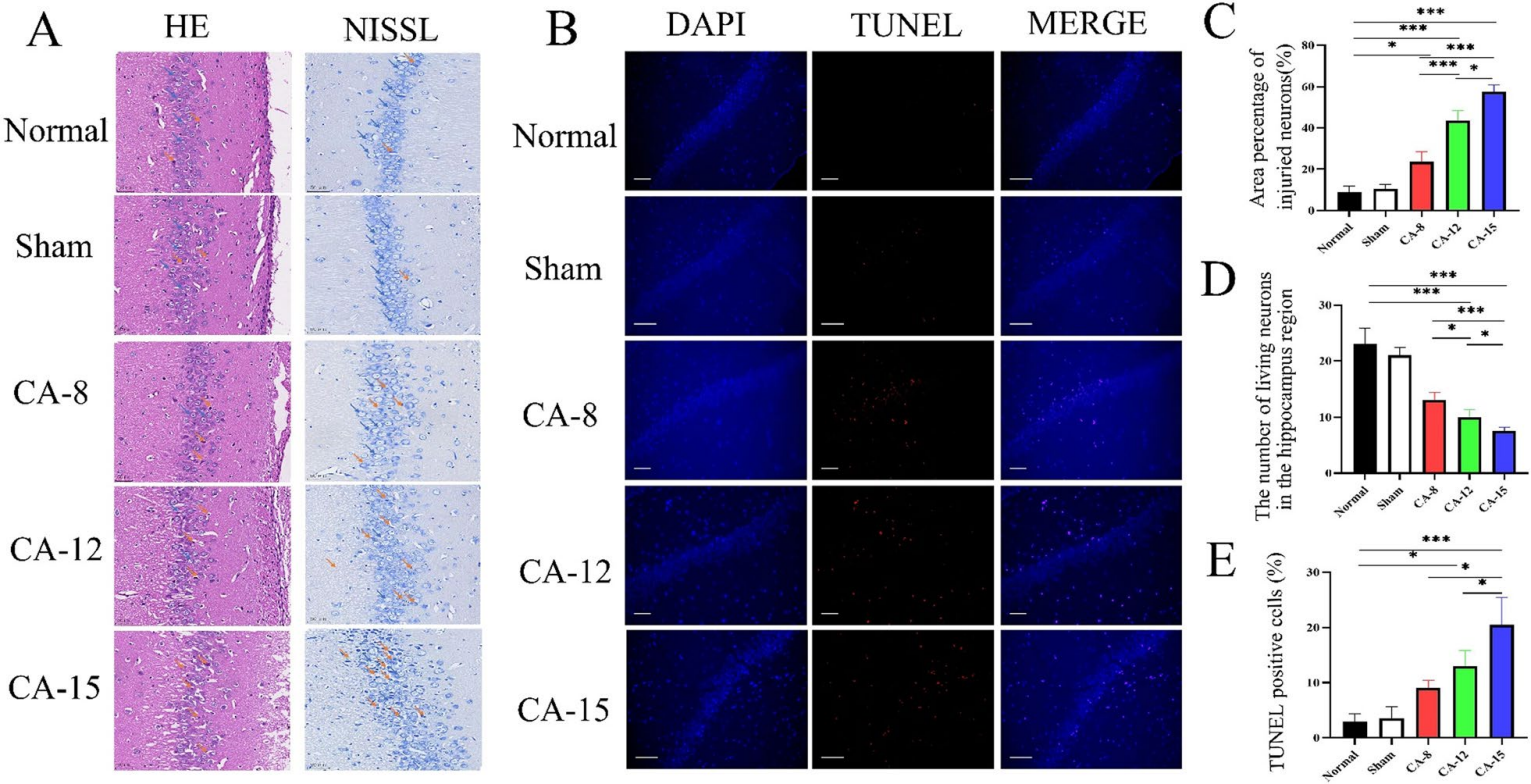
Brain histopathology. **A** HE staining and Nissl staining in the hippocampus of the rat between each group. The magnifications and scale bars were shown. **B** TUNEL staining demonstrated neural cell apoptosis in the hippocampus of the rat after cardiac arrest in each group. **C** HE staining showed that there were few injured neurons in the hippocampal CA1 area of the rats in the Normal group and the Sham group. Compared with the Normal group and the Sham group, there was a higher percentage of injured neurons in the hippocampal CA1 area of the rats after CA, and the severity was increased with duration of CA time. **D** The percentage of surviving neurons in the hippocampal CA1 area decreased with the duration of CA. **E** The percentage of TUNEL-positive neurons in the hippocampal area were increased with the duration of CA. *P < 0.05; **P < 0.01; ***P < 0.001. bar = 50 μm

HE staining and Nissl staining were used in this study to evaluate neuronal damage. HE staining of brain tissue is shown in Fig. 3A. The hippocampal neurons of the rats in the Normal group and the Sham group were arranged neatly, distributed more uniformly, with lighter nuclear staining, more uniform chromatin distribution, and normal neuronal fiber structure. Compared with the Normal group and the Sham group, the brain tissue slices of the CA group showed that the neurons in the hippocampus were arranged in a disorderly pattern, the distribution was extremely uneven, the number of layers was reduced and disordered, and the nucleus pyknosis and lysis were more serious (Fig. 3A, C).

Nissl staining was used to detect the survival of hippocampal neurons in each group. The surviving neurons in the normal group and the sham group were arranged neatly and densely, with visible nuclei, complete cytoplasm, and clear outlines. A large number of lightly stained blue Nissl bodies could be observed. At the same time, we clearly observed that with the extension of CA time, the number of normal neurons decreased, the arrangement became more irregular, and the number of surviving neurons decreased significantly (Fig. 3A, D).

TUNEL staining showed that there were few apoptotic cells in the Normal group and the Sham group, while the number of TUNEL staining positive cells in the hippocampal CA-1 area in the CA group increased significantly. With the prolonging of CA time, the number of TUNEL staining positive cells further increased, and the difference between the groups was statistically significant (Fig. 3B, E).

**Fig. 4:**
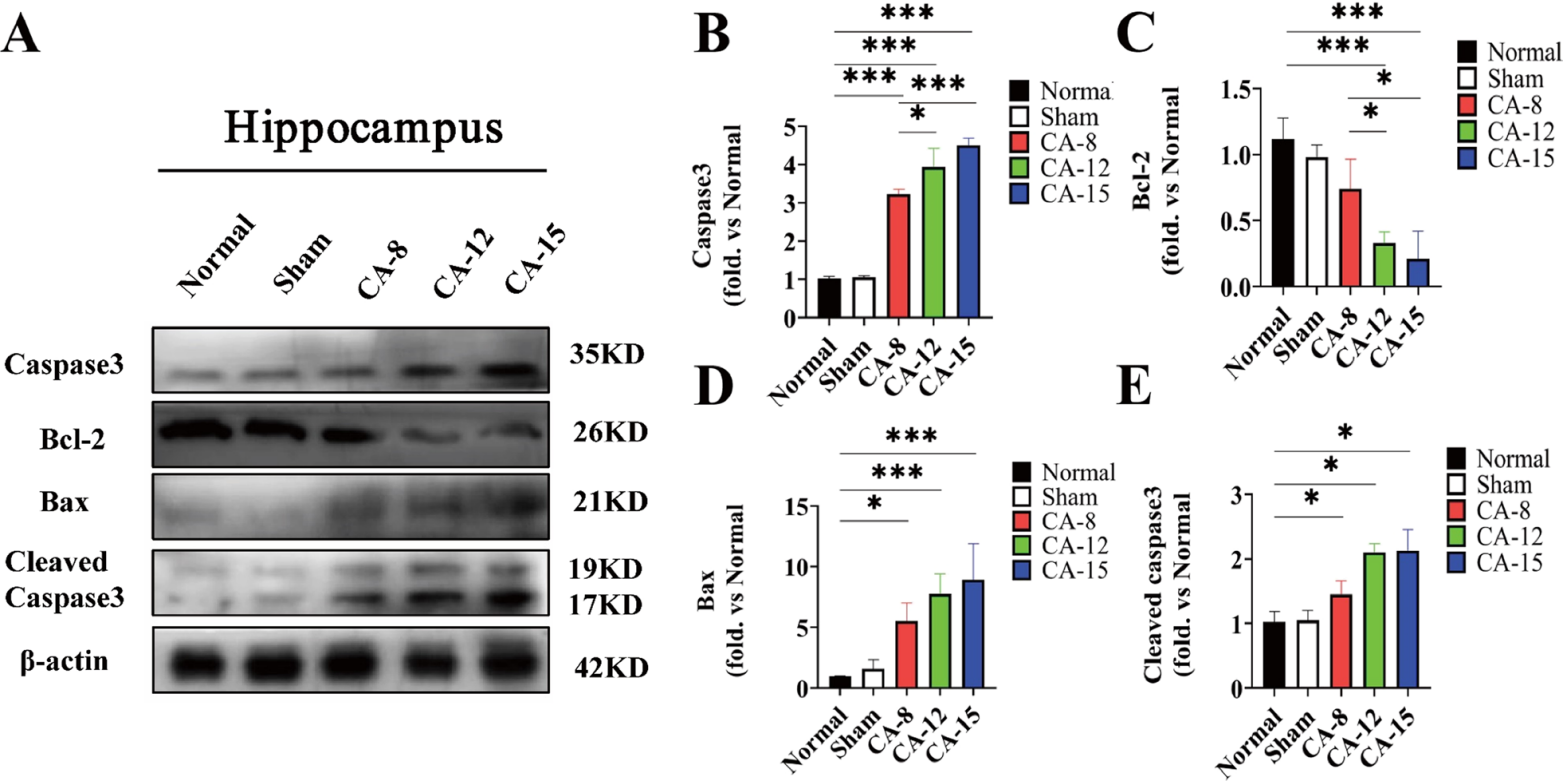
The expression of Caspase3, Bax, Bcl-2 and Cleaved caspase3 in the hippocampus of rats after cardiac arrest for different durations. **A** Five kinds of specific protein bands on the gel and their relative intensities. **B** The expression of Caspase3 increased with the duration of CA. **C** The expression of Bcl-2 decreased with the duration of CA. **D** The expression of Bax increased with the duration of CA. **E** The expression of cleaved caspase-3 increased with the duration of CA. *P < 0.05; **P < 0.01; ***P < 0.001

Consistent with the results of TUNEL staining, western blot analysis found that the expression of the proapoptotic protein Bax and the apoptotic executive protein caspase3 in the hippocampus of CA rats was significantly increased, while the expression of the antiapoptotic protein Bcl-2 was significantly reduced. Following the extension of CA time, the expression of Bax and caspase 3 also increased, while the expression of Bcl2 further decreased (Fig. 4A–E).

## Discussion

Cardiovascular diseases remain the leading cause of death worldwide. In the early 1990s, Paradis et al. pioneered the description and definition of cardiac electromechanical dissociation (EMD), which is now widely used to describe content such as PEA [15]. Most of them involved the hemodynamic status of patients with cardiac electrical activity, but no palpable pulse was observed during invasive hemodynamic monitoring of CA patients. Now, it generally refers to a clinical phenomenon in which the electrical activity of the myocardium is still present even though there is no palpable pulse. In recent years, the cause of sudden cardiac death has changed, and people know less about PEA. PEA is a common mechanism of cardiac arrest, which is defined by the results of the electrocardiogram. This is mostly due to a sudden decrease in the oxygen supply of the heart, which causes the heart to fail to maintain effective systolic and diastolic functions. In animal models of cardiac arrest caused by asphyxia, hemodynamic cessation after severe bradycardia was the most commonly used method to cause PEA-CA [16]. Studies have shown that the incidence of ventricular fibrillation-induced CA is declining, while the incidence of PEA-CA has been increasing and has gradually become a dominant condition in children and adults with out-of-hospital cardiac arrest [17]. The survival rate of PEA-CA is still very low whether it is in-hospital or out-of-hospital CA. Therefore, it is necessary to better understand its underlying mechanism and explore ways to improve the survival rate of such patients [18].

This study proved that the use of vecuronium bromide (0.1 mg/kg, IV) can successfully establish a rat CPR model of CA caused by a nonshockable rhythm. After breathing was blocked, PEA appeared at approximately 320 s, and asystole appeared at approximately 440 s. There was no VF during CA, BLS and after ROSC. The BLS time of animals in each group was relatively concentrated, and the difference within groups was not significant. In this study, the success rate of the rat PEA-CA model was 100%, while the previous VF-CA model induced by current stimulation of the myocardium had obvious uncertainty. Since the effective refractory period of rat cardiomyocytes was relatively short, if the current stimulation was not applied for more than 90 s, automatic cardioversion and ischemic preconditioning of the whole body tended to occur. If continuous electrical stimulation was applied, it easily aggravated myocardial damage and affected the success rate of ROSC. Therefore, the VF-CA model of VF induced by electrical stimulation did not easily control the duration of VF. At the same time, the BLS time of different animals was also very different and usually required a larger number of samples to achieve significant differences. In this experiment, the time for animals to present with PEA and asystole was relatively constant, and the BLS time of animals in each group was also constant. Therefore, the animal model of this experiment was easily standardized, which was beneficial for the evaluation of subsequent experimental indicators.

Post resuscitation syndrome (PRS) is also known as post-resuscitation multiple organ dysfunction syndrome (PR-MODS), which refers to multiple organ dysfunction syndrome secondary to CA-CPR. This is mainly due to the dysfunction or disorder of multiple important organs of the whole body, including the brain, heart, lung, kidney, liver, pancreas, etc., which occurs after CPR-ROSC. Most patients died of early heart failure and neurological impairment after ROSC [19–21]. Therefore, the severity of organ damage should also be an important indicator for evaluating the success of a CA model [22]. The results of this study showed that significant cardiac and neurological impairment can occur after CA for 8, 12 and 15 min in rats, and the severity was significantly related to the duration of CA. The scores of neurological functions and the indices of cardiac systolic function were also decreased significantly.

Histopathological examination revealed that the neuron cell count in the CA1 area of the brain decreased after ROSC and that the apoptotic cell counts increased. A longer duration of CA resulted in more obvious changes in the above results. Neuronal cell apoptosis after ROSC is an important mechanism of secondary brain injury. In this study, it was found that the expression of the proapoptotic proteins Bax and caspase-3 increased after ROSC, while the expression of the antiapoptotic protein Bcl-2 decreased. With the extension of CA time, this change became more obvious. In this study, the ROSC rate of CA for 8 min was 100%, the ROSC rate at CA for 12 min was 83%, and the ROSC rate at CA for 15 min was 56%. The 72-h survival rates of the three groups of rats were 100%, 58%, and 21%. These results showed that the duration of cardiac arrest directly affected the success rate of ROSC and the recovery of heart and brain functions after ROSC, which was consistent with the clinical reality.

There were some limitations in this study. First, the systolic function of the heart after ROSC cannot be continuously monitored due to the influence of the equipment, and the diastolic function after ROSC was not evaluated. Second, the score of the neurological function after ROSC adopted the classic NDS. Some of the scoring items in the NDS were affected by subjective factors, and the more refined and objectively scored items, such as open field test, water maze and other behavioral experiments, learning and memory ability evaluation may be better. Third, except for heart and brain impairment evaluation, no other organ functions were monitored.

In this study, the use of vecuronium to block breathing allowed us to successfully establish a rat CA model caused by nonshockable rhythm, which will be helpful in further studying the pathophysiological mechanism of patients after CA caused by nonshockable rhythm (Additional file 1).

## Supplementary information

**Additional file 1**. Original, full-length gel and lot images of Fig. 4A.

**Additional file 2** Video of BLS.

## Data Availability

Raw data are available from corresponding author upon reasonable request.
