# Supplementary material for: Establishment of a nonshockable rhythm cardiac arrest model caused by asphyxia
Source: BMC Cardiovasc Disord. 2022 Dec 29;22:573. doi: 10.1186/s12872-022-02996-w (PMC9798662; doi:10.1186/s12872-022-02996-w)
Supplement: Supplementary file 1 — Additional file 1. Original, full-length gel and lot images of Fig. 4A. [file 12872_2022_2996_MOESM1_ESM.pptx]

## Slide 1
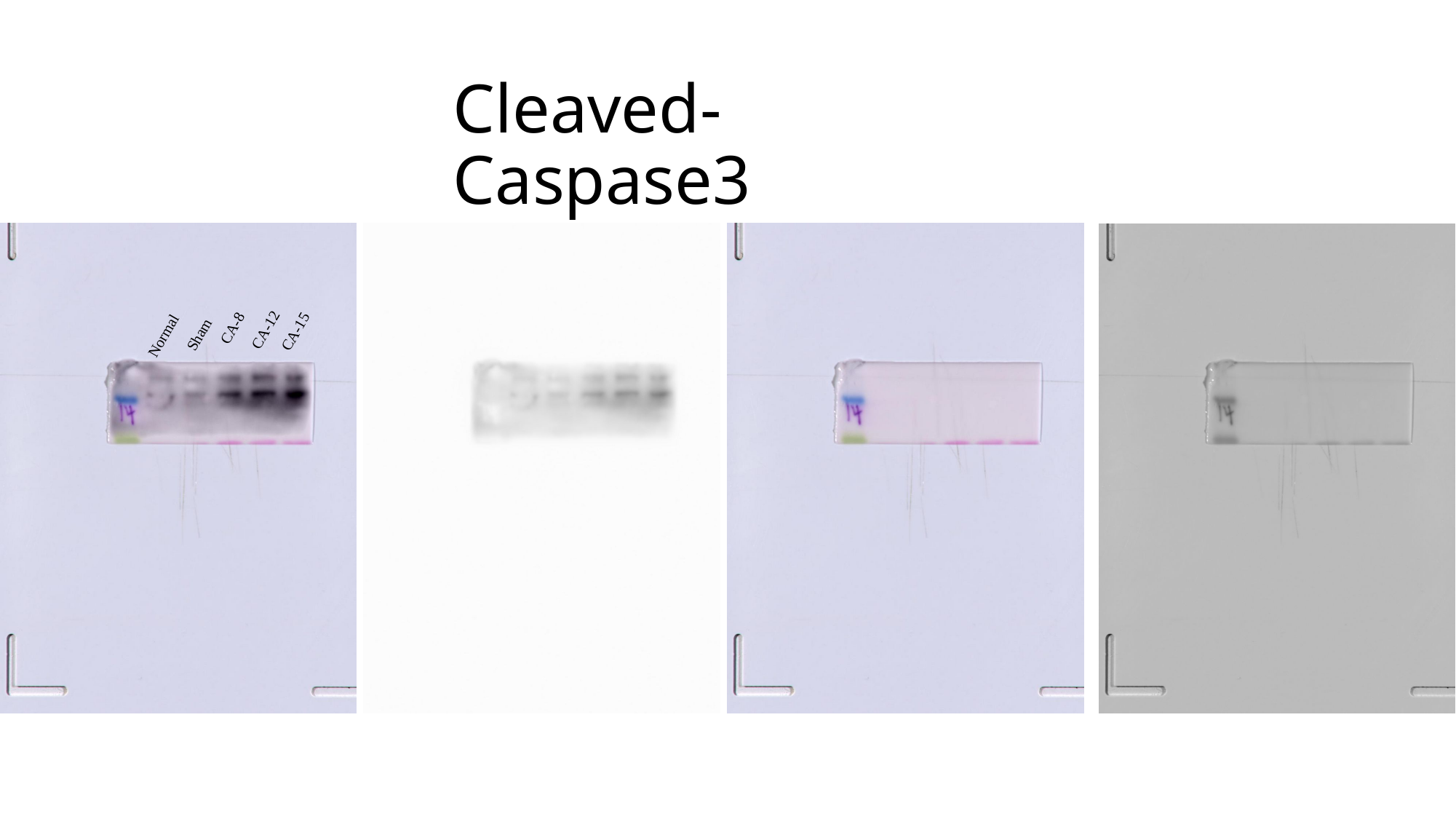

# Cleaved-Caspase3
CA-8
CA-12
CA-15
Sham
Normal

## Slide 2
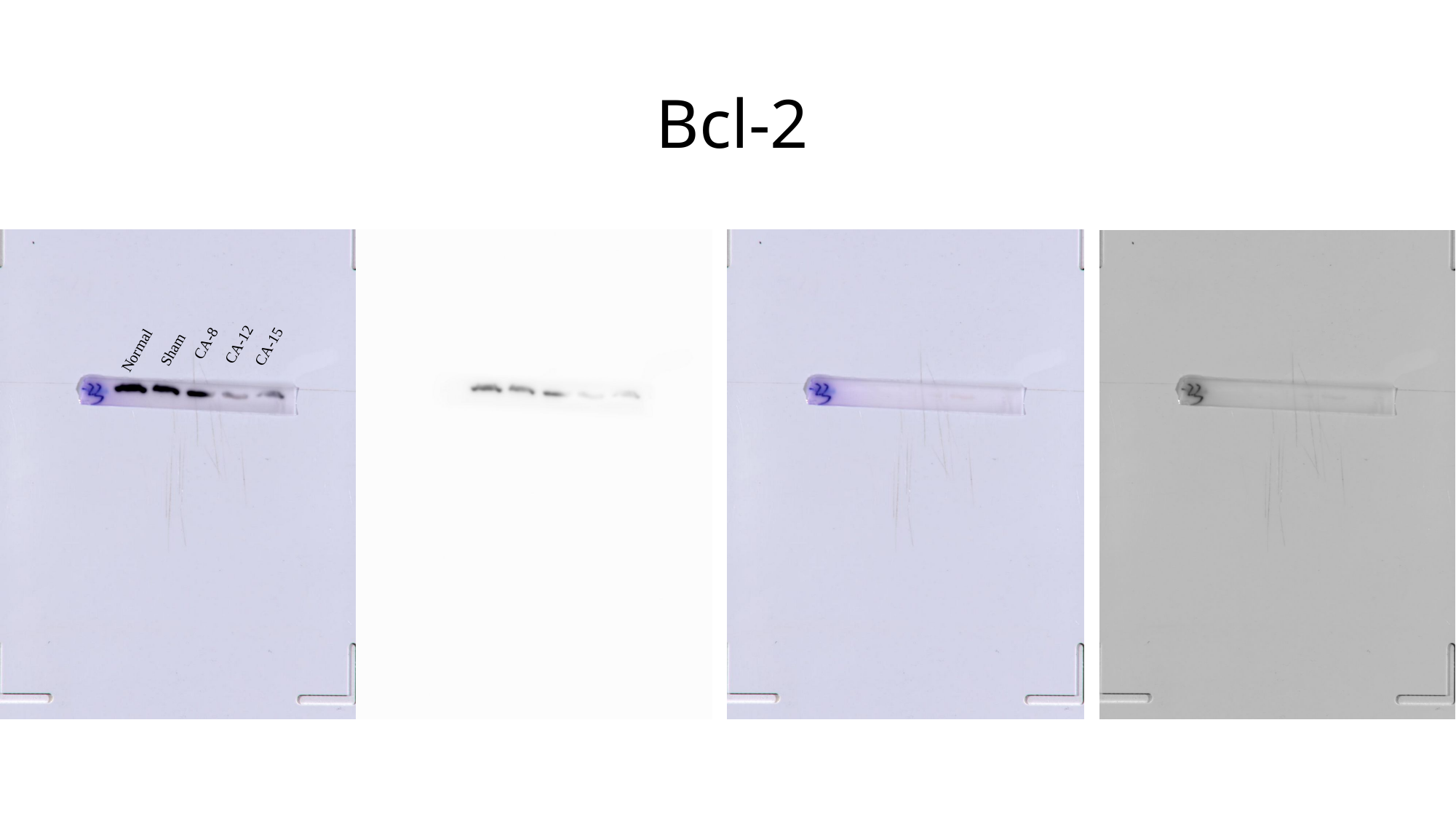

# Bcl-2
CA-8
CA-12
CA-15
Sham
Normal

## Slide 3
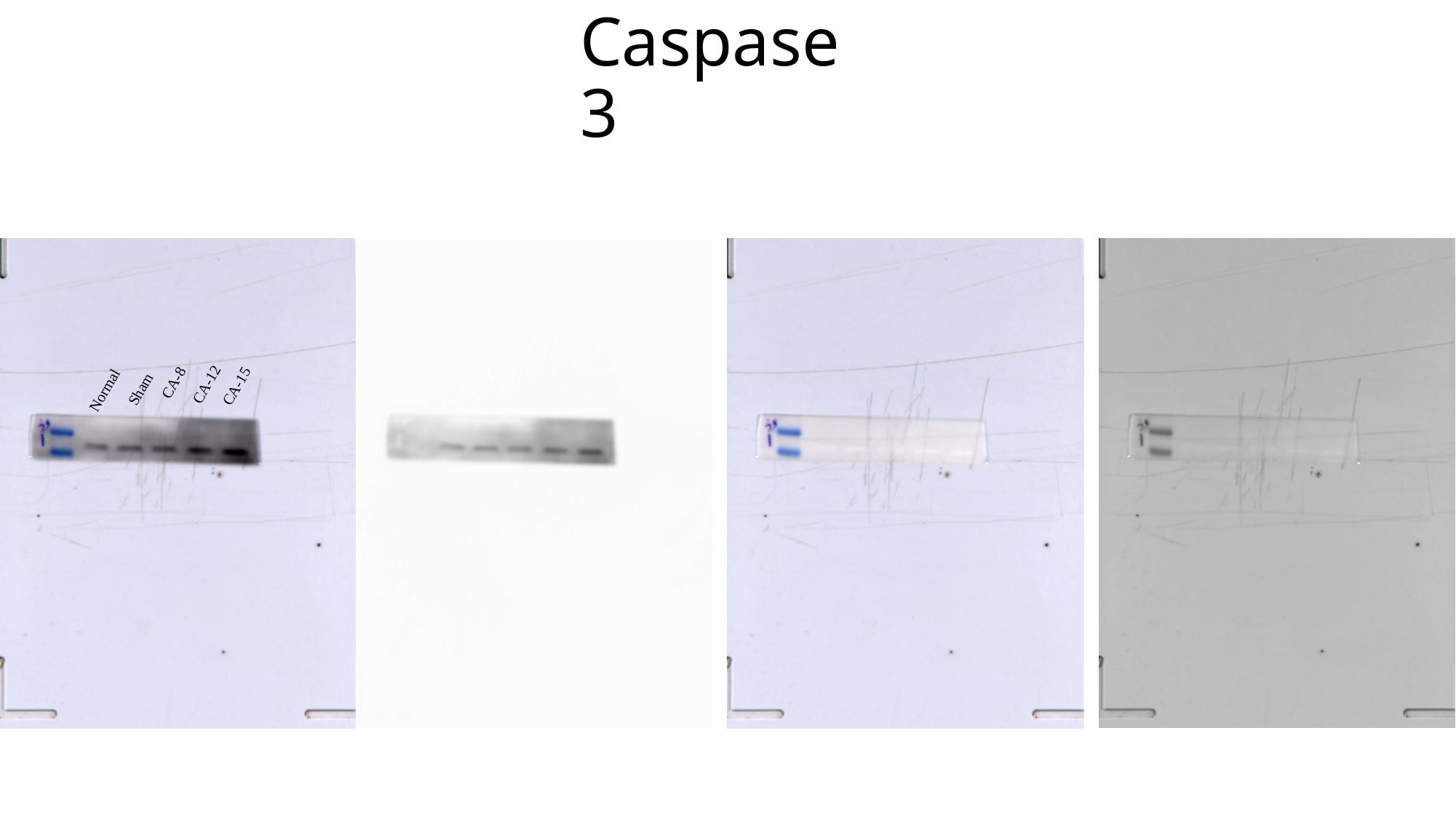

# Caspase3
CA-8
CA-12
CA-15
Sham
Normal

## Slide 4
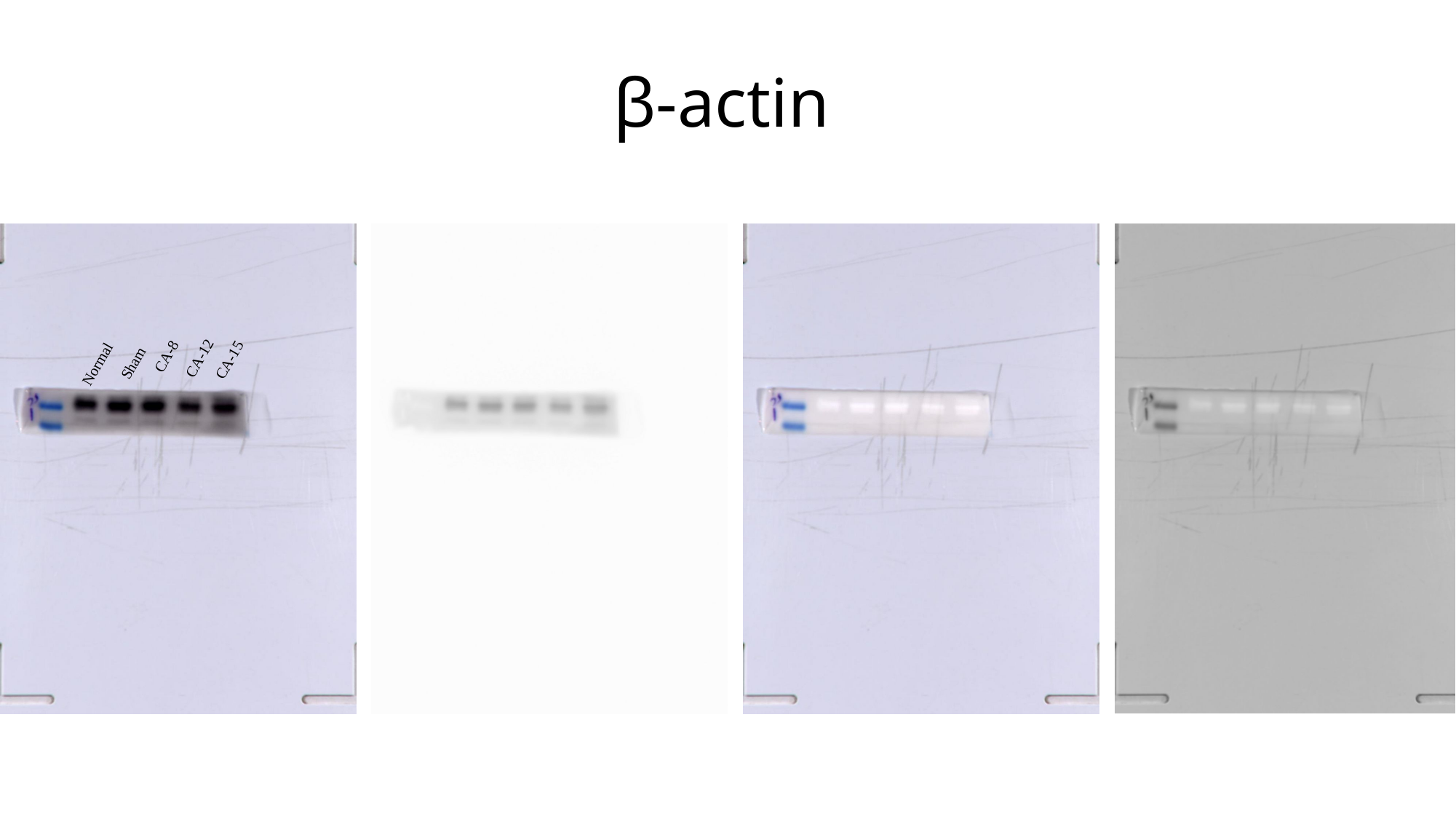

# β-actin
CA-8
CA-12
CA-15
Sham
Normal

## Slide 5
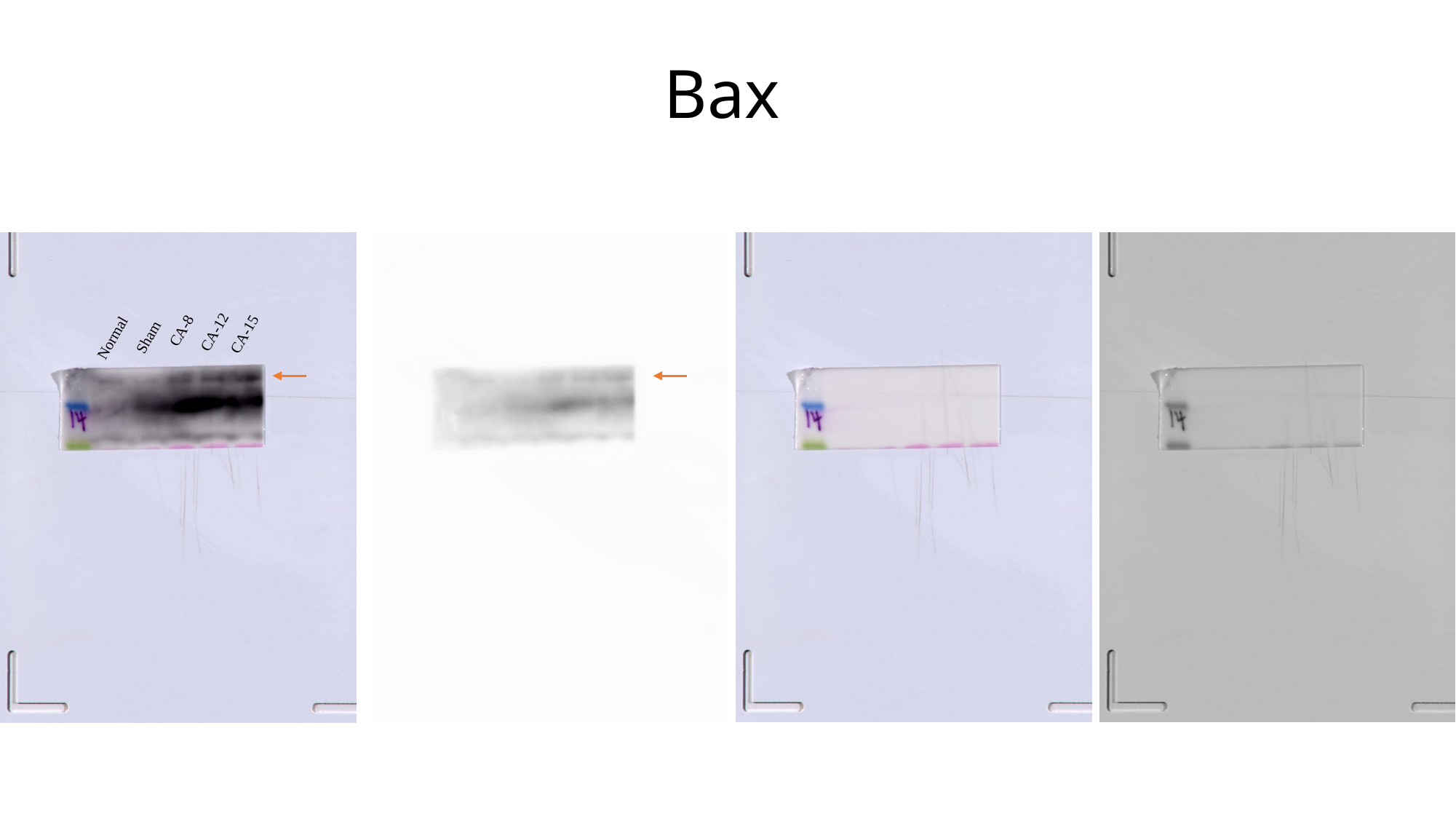

# Bax
CA-8
CA-12
CA-15
Sham
Normal
